# Extrahepatic Portal Hypertension following Liver Transplantation: a Rare but Challenging Problem

**DOI:** 10.1155/1998/81832

**Published:** 1998

**Authors:** B. Malassagne, O. Soubrane, B. Dousset, P. Legmann, D. Houssin

**Affiliations:** 1 Clinique Chirurgicale Hôpital Cochin Paris France; 2 Service de Radiologie Hôpital Cochin Paris France

## Abstract

This study reports our experience of 8 cases of extrahepatic portal hypertension after 273 orthotopic liver transplantations in 244 adult patients over a 10- year period. The main clinical feature was ascites, and the life-threatening complication was variceal bleeding. Extrahepatic portal hypertension was caused by portal vein stenosis in 6 patients, and left-sided portal hypertension in 2 patients after inadventent ligation of portal venous tributaries or portasystemic shunts. All patients with portal vein stenosis had complete relief of portal hypertension after percutaneous transhepatic venoplasty (*n*=4) or surgical reconstruction (*n*=2), after a median follow-up of 33 (range: 6–62) months. Of the 2 patients with left-sided portal hypertension, one died after splenectomy and one rebled 6 months after left colectomy. This study suggests that extrahepatic portal hypertension is a series complication after liver transplantation that could be prevented by meticulous portal anastomosis and closure of portal tributaries or portasystemic shunts to improve the  portal venous flow. However, any ligation has to be performed under ultrasound guidance to avoid inadventent venous ligations.


*HPB Surgery*, 1998, Vol. 10, pp. 357-364
Reprints available directly from the publisher
Photocopying permitted by license only

© 1998 OPA (Overseas Publishers Association)
Amsterdam B.V. Published under license
under the Harwood Academic Publishers imprint,
part of The Gordon and Breach Publishing Group.
Printed in India.

### Extrahepatic Portal Hypertension following Liver Transplantation: a Rare but Challenging Problem

B. MALASSAGNE [a], O. SOUBRANE [a,*], B. DOUSSET [a], P. LEGMANN [b] and D. HOUSSIN [a]

[a]*Clinique Chirurgicale;* [b]*Service de Radiologie, Hôpital Cochin, Paris*

*(Received 25 May 1996; In final form 22 March 1997)*

**This study reports our experience of 8 cases of extrahepatic portal hypertension after 273 orthotopic liver transplantations in 244 adult patients over a 10-year period. The main clinical feature was ascites, and the life-threatening complication was variceal bleeding. Extrahepatic portal hypertension was caused by portal vein stenosis in 6 patients, and left-sided portal hypertension in 2 patients after inadventent ligation of portal venous tributaries or portasystemic shunts. All patients with portal vein stenosis had complete relief of portal hypertension after percutaneous transhepatic venoplasty (*n*=4) or surgical reconstruction (*n*=2), after a median follow-up of 33 (range: 6-62) months. Of the 2 patients with left-sided portal hypertension, one died after splenectomy and one rebled 6 months after left colectomy. This study suggests that extrahepatic portal hypertension is a series complication after liver transplantation that could be prevented by meticulous portal anastomosis and closure of portal tributaries or portasystemic shunts to improve the portal venous flow. However, any ligation has to be performed under ultrasound guidance to avoid inadventent venous ligations.**

*Keywords:* Extrahepatic portal hypertension, orthotopic liver transplantation

Vascular complications have been a constant cause of morbidity and mortality after orthotopic liver transplantation [1, 2]. Obstruction of the portal vein or its tributaries is a rare complication leading either to acute graft failure [3] or development of extrahepatic portal hypertension. Little is known about the long-term consequences of extrahepatic portal hypertension following late portal vein obstuction [4]. Its appropriate therapeutic management remains to be determined, particularly in case of left-sided portal hypertension [5]. Until recently, vascular reconstruction was the main-stay of treatment [6-8], but nonoperative procedures, such as balloon dilatation of portal venous strictures are now available [9-14]. In this study, we report our experience of eight liver transplant recipients who developed extrahepatic portal venous hypertension paying particular attention to presenting symptoms, treatment and outcome.

### PATIENTS AND METHODS

From January 1985 to June 1995, we performed 273 orthotopic liver transplantations in 244

*Address correspondence and requests for reprints to: Olivier Soubrane, M.D., Clinique Chirurgicale, Hôpital Cochin, 27 rue du Faubourg Saint Jacques, 75014 Paris, France. Phone number: 33 1 42 34 14 34, Fax number: 33 1 43 26 56 78.

adults. Arteriography was systematically performed during pretransplant work-up in these adult patients. Orthotopic liver transplantation was performed according to the standard technique [15]. The portal vein anastomosis was always performed with running 5-0 polypropylene sutures including a "growth factor" [16]. Standard portal vein reconstruction was done between the trunks of the donor's and recipient's portal vein. In case of portal vein hypoplasia, the anastomosis was done between the graft's portal vein and the confluence of the recipient's superior mesenteric and splenic veins. In case of portal vein thrombosis a venous homograft harvested from the donor was interposed between the confluence of the recipient's superior mesenteric and splenic veins and the donor's portal vein. After reconstruction of the portal vein, spontaneous or patent surgical portasystemic shunts were systematically ligated. Doppler ultrasound studies were done daily during the posttransplant course and repeated thereafter as required by the clinical situation and once a year at least. Abnormal Doppler signals of the portal vein or its tributaries led us to perform arteriography. Transhepatic balloon dilatation was attempted first in patients with portal vein stenosis that was diagnosed more than one month after transplantation, whereas in cases of early portal vein stenosis, surgical reconstruction was performed, sometimes with concommitant biliary reconstruction. Follow-up started on the day of treatment.

### RESULTS

### Incidence

Extrahepatic portal hypertension was identified in eight patients (3%) of mean age 38±14 years (range: 20-59 years). Over the same period, five additional patients had early portal vein thrombosis with acute graft failure and were therefore excluded from this study. Indications for orthotopic liver transplantation, the status of the portal system before transplantation, and technical adjustments required for portal reconstruction during transplantation are summarized in Table I. The median period of time between transplantation and the occurrence of symptoms was 226 (range: 15-1463) days. None of these patients had evidence of ongoing liver allograft disease, as suggested by normal liver biochemistry and histology, and negative viral screening.

### Clinical Manifestations

All eight patients developed symptoms of extrahepatic portal hypertension. No asymptomatic portal obstructions were detected by Doppler ultrasound. Time span between transplantation and signs of portal hypertension are detailed in Table II. Ascites was the initial presenting symptom in seven of eight patients (Tab. II). The other patient (#7) had no ascites. Abundance of ascites required repeated paracentesis, albumin infusion and diuretics in four patients. Endoscopic examination showed that all eight patients developed esophageal or colic varices (grade 2 and 3) and four of them bled. Two patients had severe hypersplenism. Liver function tests were within normal ranges except in one patient (patient #2) who presented with severe cholestasis related to an ischemic biliary stricture.

### Mechanism Involved

All eight patients had abnormal venous Doppler signals and underwent arteriography. No arterial abnormalities were detected on arteriography. Portal vein stenosis in six patients and left-sided-portal hypertension in two were observed. The two patients with left-sided-portal hypertension had complete portal vein thrombosis before transplantation. Left-sided portal hypertension was caused by the exclusion of some part of the portal territory during portal vein reconstruction. In patient #6, portal vein thrombosis extended proximally to the confluence of splenic, superior and inferior mesenteric veins

TABLE I Pretransplant characteristics and technical adjustments in 8 (3%) liver graft recipients with postoperative extrahepatic portal hypertension

| Patients No. | Age (years) | Liver disease | Pretransplant portal status | Technical adjustments |
|---|---|---|---|---|
| 1 | 52 | Posthepatitis B cirrhosis | – | – |
| 2 | 18 | Cystic fibrosis | Spontaneous coronaroazygos shunt and thrombosed distal splenorenal shunt | Shunts taken down |
| 3 | 59 | Cryptogenic cirrhosis | Partial thrombosis of PV* | Thrombectomy; dissection to SV-SMV‡ confluence |
| 4 | 34 | Posthepatitis C cirrhosis | Patent side-to-side portacaval shunt | Shunt taken down |
| 5 | 44 | Posthepatitis C cirrhosis with hepatocellular carcinoma | Patent distal splenorenal shunt and thrombosed side-to-side portacaval shunt | Shunts taken down |
| 6 | 42 | Cryptogenic cirrhosis | – | – |
| 7 | 32 | Polyadenomatous disease | Thrombosis of PV and SV†; spontaneous splenorenal shunt | Graft portal vein anastomosis to RSCV¶; Division of the marginal colic vein between RSCV and LSCV§ to add lenght |
| 8 | 20 | Autoimmune hepatitis | Thrombosis of PV and SV; patent spontaneous coronaroazygos and splenorenal shunts | Venus jump graft to SMV; shunts taken down |

*PV, portal vein.

†SV, splenic vein.

‡SMV, superior mesenteric vein.

¶RSCV, right superior colic vein.

§LSCV, left superior colic vein.

TABLE II Cause, clinical presentation, treatment and outcome of extrahepatic portal hypertension in 8 (3%) liver graft recipients

| Patients No. | Cause | Clinical Presentation (days after OLT*) | | | | Treatment (days after OLT*) | Outcome after treatment |
|---|---|---|---|---|---|---|---|
| | | Ascites | Varices | Variceal Bleeding | Hypersplenism | | |
| 1 | PVS[†] | +(15) | +(25) | +(25) | | Surgery (30) | Alive and well (62 months) |
| 2 | PVS | +(233) | +(240) | | +(233) | Surgery (250) | Alive and well (14 months) |
| 3 | PVS | +(219) | +(225) | +(251) | | Sclerotherapy (251)/Angioplasty (260) | Alive and well (59 months) |
| 4 | PVS | +(1440) | +(1440) | | | Angioplasty (1449) (two courses) | Alive and well (51 months) |
| 5 | PVS | +(90) | +(97) | | | Angioplasty (107) | Died of HCC[¶] recurrence (15 months) |
| 6 | PVS | +(720) | +(725) | | | Angioplasty (739) | Alive and well (6 months) |
| 7 | LSPH[‡] | | +(colic) (1463) | +(1463) | | Left colectomy (1677) | Alive with recurrent bleeding (32 months) |
| 8 | LSPH | +(20) | +(gastric) (34) | +(34) | +(30) | Splenectomy (41) | Died of sepsis (50 days) |

*OLT, orthotopic liver transplantation.

†PVS, portal vein stenosis.

‡LSPH, left-sided portal hypertension.

¶HCC, hepatocellular cacinoma.

with development of a cavernomatous network. The largest vein available for reconstruction was the right superior colic vein. Since the marginal colic vein had to be divided to add length, inferior and superior mesenteric beds were separated and the inferior mesenteric bed was thus excluded from the portal drainage. In patient #7, who had a thrombosed splenic vein before transplantation, the erroneous ligation of both the coronary vein and a spontaneous splenorenal shunt resulted in exclusion of the splenic bed.

### Treatment

Results are summarized in Table II.

Portal vein stenosis was treated by percutaneous balloon dilatation in four patients. The pressure gradient was 7, 7, 10 and 5 mm Hg before dilation and completely disappeared after the procedure. There was no complication. Repeat endoscopic examination showed a variceal recurrence in one patient six months after the procedure requiring a second course of angioplasty. At a median follow-up of 33 (range: 6–59) months those patients had no more sign of portal hypertension. Surgery was preferred to transhepatic venoplasty in two patients with a portal vein stenosis occurring either too early after transplantation (i.e. within the first month) or in association with an anastomotic biliary stricture. In the latter case, the length of ischemic biliary stricture precluded transhepatic dilatation. Surgery consisted of excision of the previous anastomosis and redoanastomosis without graft interposition. Both patients recovered successfully without recurrence 62 and 14 months after reconstruction.

Left-sided portal hypertension was corrected by the resection of the excluded part of the portal territory. Patient #7, who had an exclusion of the inferior mesenteric area, was treated by a left colectomy and recovered uneventfully. However he bled again six months later from rectal varices. The patient #8 with portal hypertension within the splenic area was treated by a splenectomy. Extreme technical difficulties have been encountered during this operation because of portal hypertension, post-transplant adhesions and splenic enlargement. This patient had a severe postoperative pancreatitis and died 50 days later from intrabdominal sepsis and multi-organ failure.

### DISCUSSION

Extrahepatic portal hypertension following liver transplantation has not been studied extensively in the past since complications of the portal system are uncommon in liver transplant recipients [1, 7, 17, 18].

Ascites was the first clinical symptom in all but one patient. In this series, four patients experienced variceal bleeding which is a life-threatening complication. Thus, even though post-transplant ascites is frequent after liver transplantation, its occurrence after a period of no ascites in patients with normal liver biochemistry and histology is strongly suggestive of portal vein obstruction. This should lead one to perform adequate imaging studies in order to expeditiously correct the portal obstruction before the development of varices and possible bleeding.

Although Doppler ultrasound identified the cause of portal obstruction in eight patients, angiography was also performed to provide a complete mapping of the portal system [19], and to detect arterial abnormalities.

Left-sided portal hypertension occurred in two patients with pretransplant portal vein thrombosis. Both of whom required technical adjustments at the time of transplantation. This has been previously reported by Stevenson *et al.* [20]. In these patients, portal vein tributaries or portasystemic shunts were erroneously ligated and the splenic or the inferior mesenteric areas were excluded from the portal system. These cases suggest that distal portasystemic shunts should not be routinely ligated: ligation is only indicated if closure is needed to increase the portal venous flow to the graft [21–23]. In this setting, intraoperative Doppler ultrasound may be particularly helpful in measuring the portal vein flow before and after clamping of the shunt. Left-sided portal hypertension was treated by surgical resection of the excluded portal area [5]. Of the two patients, one died of acute pancreatitis after a difficult splenectomy, and the second experienced a rebleed from rectal varices six months after a left colectomy. Definitive treatment of the latter patient was not prompt due to delayed localization of the bleeding site. This was retrospectively confirmed by bleeding recurrence from rectal varices, six months after surgery. Such resection of portal areas for the treatment of left-sided portal hypertension has never been previously described in liver transplant recipients. The other reported case of left-sided portal hypertension was treated with a venous bypass from the splenic to the renal vein [20]. In our patients, complete thrombosis of the splenic or inferior mesenteric veins precluded this option as well as makeshift shunts between the splenic or inferior mesenteric veins and the portal or the left renal vein. Since left-sided portal hypertension was due to ligation of portal tributaries or portasystemic shunts, careful review of the angiogram could have helped avoid this severe complication.

In this series, surgery was reserved for those patients who had a contraindication to transhepatic balloon dilatation for correction of post-transplant portal vein stenosis. The results of this study suggest that transhepatic angioplasty is a safe and effective procedure [9–14] with satisfactory long-term results. However, this technique is not without possible risks in the early posttransplantation period. Moreover, stenosis of the portal vein may reoccur; therefore, close postoperative surveillance is indicated, and if necessary, repeat balloon angioplasty. In this series, portal vein stenosis occurred at different times (from 25 days to 1440) in the posttransplantation period. Thus the causes of stenosis were likely to be different from patient to patient. However, it is well-accepted that portal vein stenosis is mostly due to technical mistakes during portal vein anastomosis. Thus, performance of a wide anastomosis with proximal dissection to the confluence of the splenic and superior mesenteric veins may be recommanded to prevent stenosis when the trunk of the portal vein within the porta hepatitis is hypoplastic. In other cases when the portal vein trunk is adequate, anastomosis may be performed at a distance from the pancreas. Moreover prevention of portal vein stenosis should lead to a substantial reduction in the incidence of posttransplantation extrahepatic portal hypertension.

In conclusion, extrahepatic portal hypertension may be a grave complication after liver transplantation, particularly when caused by left-sided portal hypertension. Attention to technique during the portal vein anastomosis and avoiding the routine ligation of portal vein tributaries will help prevent the occurrency of such extrahepatic portal hypertension.

### *Acknowledgement*

We thank Dr William Inabnet for reviewing the manuscript.

### *References*

[1] Langnas, A. N., Marujo, W., Stratta, R. J., Wood, P. and Shaw, B. W. (1991). Vascular complications after orthotopic liver transplantation. *American Journal of Surgery*, **161**, 76–82.

[2] Busutill, R. W., Shaked, A., Millis, J. M., Jurim, O., Colquhoun, S. D., Shackleton, C. R., Nuesse, B. J., Csete, M., Goldstein, L. I. and McDiarmid, S. V. (1994). One thousand liver transplants. The lessons learned. *Annals of Surgery*, **219**, 490–499.

[3] Langnas, A. N., Marujo, W. C., Stratta, R. J., Wood, R. P., Li, S. and Shaw, B. W. (1991). Vascular complications following orthotopic liver transplantation: outcome and role of urgent liver revascularization. *Transplantation Proceedings*, **23**, 1484–1486.

[4] Wozney, P., Zajko, A. B., Bron, K. M., Point, S. and Starzl, T. E. (1986). Vascular complications after liver transplantation. A 5-Year experience. *American Journal of Roentgenology*, **147**, 657–663.

[5] Turrill, F. L. and Mikkelsen, W. P. (1969). "Sinistral" (left sided) extrahepatic portal hypertension, *Archives of Surgery*, **99**, 365–368.
[6] Zajko, A. B., Bron, K. M., Starzl, T. E., Van Thiel, D. H., Carlton Gartner, J., Iwatsuki, S., Shaw, B. W., Zitelli, B. J., Jeffrey Malatack, J. and Urbach, A. H. (1985). Angiography of liver transplantation patients. *Radiology*, **157**, 305–311
[7] Lerut, J., Tzakis, A. G., Bron, K., Gordon, R. D., Iwatsuki, S., Esquivel, C. O., Makowka, L., Todo, S. and Starzl, T. (1987). Complications of venous reconstruction in human orthotopic liver transplantation. *Annals of Surgery*, **205**, 404–414.
[8] Scantlebury, V. P., Zajko, A. B., Esquivel, C. O., Marino, I. R. and Starzl, T. E. (1989). Successful reconstruction of late portal vein stenosis after hepatic transplantation. *Archives of Surgery*, **124**, 503–505.
[9] Ullacker, R., Alves, M. A., Cantisani, G. C., Souza, H. P., Wagner, J. and Moraes, L. F. (1985). Treatment of portal vein obstruction by percutaneous transhepatic angioplasty. *Gastroenterology*, **88**, 176–180.
[10] Raby, N., Karani, J., Thomas, S., O'Grady, J. and Williams, R. (1991). Stenoses of vascular anastomoses after hepatic transplantation: treatment with balloon angioplasty. *American Journal of Roentgenology*, **157**, 167–171.
[11] Rollins, N. K., Sheffield, E. G. and Andrews, W. S. (1992). Portal vein stenosis complicating liver transplantation in children: percutaneous transhepatic angioplasty. *Radiology*, **182**, 731–734.
[12] Legmann, P., Martin Bouyer, Y., Tudoret, L., Limot, O., Calmus, Y., Houssin, D. and Bonnin, A. (1993). Portal vein stenosis after liver transplantation: treatment with percutaneous transhepatic angioplasty. *European Radiology*, **3**, 371–375.
[13] Azoulay, D., Castaing, D., Ahchong, K., Adam, R. and Bismuth, H. (1993). A minimally invasive approach to the treatment of stenosis of the portal vein after hepatic transplantation. *Surgery Gynecology and Obstetrics*, **176**, 599–601.
[14] Zajko, A. B., Sheng, R., Bron, K. M., Reyes, J., Nour, B. and Tzakis, A. (1994). Percutaneous transluminal angioplasty of venous anastomotic stenoses complicating liver transplantation: Intermediate-term results. *Journal of Vascular and Interventional Radiology*, **5**, 121–126.
[15] Starzl, T. E., Iwatsuki, S., Esquivel, C. O., Todo, S., Kam, I., Lynch, S., Gordon, R. D. and Shaw, B. W. Jr. (1985). Refinements in the surgical technique of liver transplantation. *Semin Liver Dis.*, **5**, 349–356.
[16] Starzl, T. E., Iwatsuki, S. and Shaw, B. W. Jr. (1984). A growth factor in fine vascular anastomoses. *Surgery Gynecology and Obstetrics*, **159**, 164–165.
[17] Cherqui, D., Duvoux, C., Rahmouni, A., Rotman, N., Dhumeaux, D., Julien, M. and Fagniez, P. L. (1993). Orthotopic liver transplantation in the presence of partial or total portal vein thrombosis: problems in diagnosis and management. *World Journal of Surgery*, **17**, 669–674.
[18] Davidson, B. R., Gibson, M., Dick, R., Burroughs, A. and Rolles, K. (1994). Incidence, risk factors, management, and outcome of portal vein abnormalities at orthotopic liver transplantation. *Transplantation*, **57**, 1174–1177.
[19] Orons, P. D. and Zajko, A. B. (1995). Angiography and interventional procedures in liver transplantation. *Radiologic Clinics of North America*, **33**, 541–558.
[20] Stevenson, W. C., Sawyer, R. G. and Pruett, T. L. (1992). Recurrent variceal bleeding after liver transplantation-Persistent left-sided portal hypertension. *Transplantation*, **53**, 493–495.
[21] Boillot, O., Houssin, D., Santoni, Ph ,Ozier, Y., Matmar, M. and Chapuis, Y. (1991). Liver transplantation in patients with surgical portasystemic shunt. *Gastroentérologie Clinique et Biologique*, **15**, 876–880.
[22] Cherqui, D., Panis, Y., Gheung, P., Duvoux, N., Rotman, N., Golli, M., Dhumeaux, D., Julien, M. and Fagniez, P. L. (1993). Spontaneous portosystemic shunts in cirrhotics: implications for orthotopic liver transplantation. *Transplantation Proceedings*, **25**, 1120–1121.
[23] Fujimoto, M., Moriyasu, F., Nada, T., Suginoshita, Y., Ito, Y., Nishikawa, K., Someda, H., Okuma, M., Inomata, Y., Ozaki, N., Tanaka, K. and Yamaoka, Y. (1995). Influence of spontaneous portasystemic collateral pathways on portal hemodynamics in living-related liver transplantation in children. *Transplantation*, **60**, 41–45.

### COMMENTARY

The paper by Malassagne and co-workers summarizes their experience with postoperative portal venous complications after orthotopic liver transplantation. This experience is based on 273 liver transplantations performed over a period of 10 years. They experienced a 3 percent extrahepatic portal venous stenosis rate. This is roughly within the range of the cited literature. It shows that portal vein stenosis after liver transplantation is rare but its treatment needs an individualized approach.

The authors' experience with transhepatic venoplasty goes along with the reported experience in the literature as cited in the article. This modality, however, is only a choice in patients more than 4 weeks after liver transplantation. The higher rate of patients in this report with late stenosis is surprising and discordant with the literature. For early portal vein stenosis the best treatment is prevention. This should be achievable by always using a generous growth factor for a wide anastomosis and performing intraoperative ultrasound to evaluate portal venous flow. The routine use of these techniques should prevent most portal vein stenosis, or at least diagnose them early. Thus, the development of ascites or even bleeding varices should be well below 1% in the transplanted population.

It also seems to be important to stress again the use of Doppler ultrasound during the operation when ligating portal tributaries. This method, often used to improve portal venous flow after transplantation can only be done safely under ultrasound guidance and respecting alternative venous drainage pathways of the respective area.

Christoph E. Broelsch
Wolfgany Trudo Knoefel

